# Nerve tolerance to high-dose-rate brachytherapy in patients with soft tissue sarcoma: a retrospective study

**DOI:** 10.1186/1471-2407-5-79

**Published:** 2005-07-19

**Authors:** Tadahiko Kubo, Takashi Sugita, Shoji Shimose, Toshihiro Matsuo, Ken Hirao, Hiroaki Kimura, Masahiro Kenjo, Mitsuo Ochi

**Affiliations:** 1 Departments of Orthopaedic Surgery, Graduate School of Biomedical Sciences, Hiroshima University, Hiroshima, Japan; 2 Department of Orthopaedic Surgery, Hiroshima Prefecture Hospital, Hiroshima, Japan; 3 Department of Rehabilitation, Hiroshima University Hospital, Hiroshima, Japan; 4 Departments of Radiology, Graduate School of Biomedical Sciences, Hiroshima University, Hiroshima, Japan

## Abstract

**Background:**

Brachytherapy, interstitial tumor bed irradiation, following conservative surgery has been shown to provide excellent local control and limb preservation in patients with soft tissue sarcomas (STS), whereas little is known about the tolerance of peripheral nerves to brachytherapy. In particular, nerve tolerance to high-dose-rate (HDR) brachytherapy has never been properly evaluated. In this study, we examined the efficacy and radiation neurotoxicity of HDR brachytherapy in patients with STS in contact with neurovascular structures.

**Methods:**

Between 1995 and 2000, seven patients with STS involving the neurovascular bundle were treated in our institute with limb-preserving surgery, followed by fractionated HDR brachytherapy. Pathological examination demonstrated that 6 patients had high-grade lesions with five cases of negative margins and one case with positive margins, and one patient had a low-grade lesion with a negative margin. Afterloading catheters placed within the tumor bed directly upon the preserved neurovascular structures were postoperatively loaded with Iridium-192 with a total dose of 50 Gy in 6 patients. One patient received 30 Gy of HDR brachytherapy combined with 20 Gy of adjuvant external beam radiation.

**Results:**

With a median follow-up of 4 years, the 5-year actuarial overall survival, disease-free survival, and local control rates were 83.3, 68.6, and 83.3%, respectively. None of the 7 patients developed HDR brachytherapy-induced peripheral neuropathy. Of 5 survivors, 3 evaluable patients had values of motor nerve conduction velocity of the preserved peripheral nerve in the normal range.

**Conclusion:**

In this study, there were no practical and electrophysiological findings of neurotoxicity of HDR brachytherapy. Despite the small number of patients, our encouraging results are valuable for limb-preserving surgery of unmanageable STS involving critical neurovascular structures.

## Background

Success in the management of soft tissue sarcomas (STS) is often limited by the extension of lesions to neurovascular structures, because of the difficulty in dissecting the neurovascular bundle from the tumor without compromising the function and local recurrence of residual lesions. Patients with STS involving or extending to neurovascular structures may be sometimes advised to undergo an amputation. In an effort to preserve limbs, conservative surgery with various modalities such as nerve resection with or without nerve grafts, irradiation, and hyperthermia for preservation of peripheral nerves has been reported [1-4]. Brachytherapy, interstitial tumor bed irradiation, following conservative surgery has been shown to provide excellent local control and limb preservation in patients with STS, whereas little is known about the tolerance of peripheral nerves to brachytherapy. The placement of afterloading catheters directly upon neurovascular structures to irradiate such lesions appropriately subjects them to high dose radiation. The American Brachytherapy Society suggested that brachytherapy, especially high-dose-rate (HDR) brachytherapy, should be used with caution in such situations [5].

We treated 7 patients with conservative surgical resection and placement of afterloading catheters directly upon these critical structures followed by fractionated HDR brachytherapy. The purpose of this study was to evaluate the efficacy and radiation neurotoxicity of HDR brachytherapy in patients with STS in contact with neurovascular bundles.

## Methods

### Patients

Between 1995 and 2000, thirty patients with STS were treated in our institute with limb salvage surgery followed by fractionated HDR brachytherapy, after obtaining informed consent from the patients or their guardians. Among them, 7 patients with STS involving the neurovascular bundle were enrolled in this study. The median age at the time of the surgery was 53 years (range, 15–84). There were 4 males and 3 females. All of the 7 patients presented with primary tumors without distant metastasis. Three patients had malignant fibrous histiocytoma, two patients had myxoid liposarcoma, one patient had synovial sarcoma, and one had extraskeletal chondrosarcoma. The median tumor size, defined as the maximum diameter of the tumor at pathological analysis, was 9.4 cm (range, 5.5–13.0 cm). Three patients received perioperative systemic therapy, which mainly consisted of ifosfamide and doxorubicin. The clinical and pathological characteristics of the patients are listed in Table 1.

### Surgery and HDR brachytherapy

All of the STS involving or adjacent to neurovascular structures were able to be marginally resected with careful dissection of these critical structures. The pathological examination of surgical specimens demonstrated that there were no gross residual lesions and one microscopic positive margin, which was defined as tumor cells present at the resection margins. A microscopically negative margin was defined as no tumor cells at the margins. In all 6 lesions with negative margins, tumor cells were seen within 1 mm from the margins at the neurovascular bundle (Table 2).

The brachytherapy technique has been previously described [5,6]. In brief, the technique of brachytherapy used afterloading catheters placed intraoperatively in the tumor bed as a single plane implant. The afterloading catheters with a 1 cm spacing in between were placed within the surgical bed directly upon the preserved neurovascular bundles. The catheters were located either parallel or vertical to the limb axes. The afterloading catheters were fixed in position in the target region using absorbable sutures and secured to the skin at the catheter exit site with buttons. Postoperative localization films, along with opaque dummy sources inserted into each individual catheter, were taken to confirm the position of the catheters and for contouring and HDR brachytherapy planning. The three-dimensional treatment planning was performed by the Nucletron PLATO planning station (Nucletron Corp., Columbia, MD) according to the International Commission on Radiation Units and Measurements (ICRU) Repost 58 [7]. The clinical target volume (CTV) was defined by expanding the resected tumor bed by 0.5 cm margins. The planning target volume (PTV) was defined by expanding the CTV by 1.0 cm margins on the catheter plane. The PTV ranged from 52 to 134 cm^3 ^(mean, 76). Multiple dose prescription points (5 mm from the source axis) were defined in accordance with the each dwell position. The prescribed dose at the periphery of the PTV was 5 Gy per fraction. The PTV was expected to cover the whole dissected portion of the nerve because the width of the nerve was less than 5 mm in all patients. Seven to ten days after the implantation, the catheters were loaded with nominal 10 Curie Iridium-192 using Microselectron-HDR (Nucletron Corp., Columbia, MD). The total dose was 50 Gy, administered as 5 Gy per fraction. Treatments were given twice a day over 5 days with a minimum of 6 hours between fractions. However, Patient 5 with low-grade extraskeletal chondrosarcoma received 30 Gy of HDR brachytherapy as a boost combined with 20 Gy of adjuvant external beam radiotherapy (EBRT) (Table 2).

### Electrophysiological study

Motor nerve conduction velocity (MCV) of peripheral nerves was measured by standardized techniques using Nicolet VikingQuest (Nicolet Biomedical Inc., Madison, WI) at the latest follow-up. In brief, for MCV of the median nerve, supramaximal stimuli were applied to the elbow, and the compound muscle action potential was recorded from surface electrodes placed over the abductor pollicis brevis muscle. To evaluate the MCV of the sciatic nerve, F-wave conduction velocity (FWCV) of the tibial nerve was used, according to Kimura et al [8]. F responses were recorded with surface electrodes over the abductor hallucis, following supramaximal stimulation at the ankle. A total of 16 F-waves were recorded and minimal F-wave latency was determined.

### Follow-up and statistical analysis

The time of follow-up was calculated from the date of the operation. All survival analyses were evaluated with the Kaplan-Meier product-limit method. The median follow-up period was 4 years (range, 13–77 months).

## Results

### Oncological outcomes

There was one local failure and one distant failure during patients' clinical courses (Table 2). Patient 2 developed a local recurrence outside the PTV of brachytherapy 25 months postoperatively. This failure was successfully salvaged with complete resection of the recurrent lesion and 50 Gy of adjuvant EBRT, but the patient experienced a femoral shaft fracture 32 months later. Patient 6 died of progressive pulmonary metastases but with local control maintained. Patient 7 died of heart problems. The other four patients survived and continued to be disease-free. The 5-year actuarial overall survival, disease-free survival, and local control rates were 83.3, 68.6, and 83.3%, respectively.

### Complications

Two patients had nerve-associated complications at the time of follow-up (Table 2). Patient 1 developed an immediate postoperative palsy of the anterior interosseous nerve prior to brachytherapy. Patient 5 had experienced sensory loss of the tibial nerve since before treatment, possibly caused by the lesion compressing the tibial nerve. In both cases, these complications have been improving. Therefore, there was no practical evidence of HDR brachytherapy-induced neurotoxicity. Complications other than nerve-associated problems included the above-mentioned femoral shaft fracture.

To further investigate the subclinical nerve damage by HDR brachytherapy, MCV studies were carried out. Of five survivors, two patients who had radiation-unrelated neuropathy were excluded. The MCV value of the median nerve and FWCV values of the tibial nerves (for MCV of the sciatic nerves) were in the normal range [8,9], consistent with our clinical findings (Table 3).

## Discussion

Brachytherapy has many theoretical and practical advantages, compared to EBRT. The shorter overall treatment time offers the patient conveniences and reduces the financial cost of treatment [10], in contrast to the standard 7–8 week course of EBRT. The rapid dose fall-off of brachytherapy spares more surrounding normal tissues [5].

Several large clinical studies have demonstrated the efficacy of conventional low-dose-rate (LDR) brachytherapy as an adjuvant therapy for STS. LDR brachytherapy provided adequate local control and acceptable morbidity compared favorably with those of EBRT [11,12]. According to prospective randomized trials, adjuvant brachytherapy improves local control after complete resection of soft tissue sarcomas. This improvement is limited to patients with high-grade histopathology [6,13]. Following these reports, we have been treating only high-grade soft tissue sarcoma with adjuvant brachytherapy after 1996. In this study, Patient 5 with a low-grade lesion received adjuvant brachytherapy in 1995. Regarding the direct effect of LDR brachytherapy on peripheral nerves, it has been reported that none of the 38 patients with STS involving the neurovascular bundle developed radiation neuropathy after receiving conservative tumor resection and cumulative doses less than 9,000 cGy of LDR brachytherapy combined with or without EBRT [14]. A histological and electrophysiological study using rabbits observed that irradiation by iridium-192 LDR brachytherapy to doses up to 13,000 cGy on the carotid-sheath contents including the vagus nerve was well tolerated [15].

Compared to LDR brachytherapy the use of HDR is an attractive alternative, because this technique allows treatment to be given in minutes instead of days, eliminating the radiation hazards and prolonged hospital stays associated with LDR. HDR brachytherapy is expected to replace traditional LDR brachytherapy, although there is limited experience in the use of HDR, both in terms of the duration and the number of cases, compared to LDR [16,17]. Further clinical data are needed to determine the specific role of HDR in the management of STS. There are no clinical reports, which properly evaluate HDR brachytherapy-inducing neuropathy, and no experimental studies on nerve tolerance to HDR brachytherapy. In general, HDR brachytherapy is believed to carry a large risk of nerve damage. It has been proposed that a layer of gel-foam or muscle should be interposed between the catheters and the neurovascular bundles [18,19]. Nevertheless, the results of our study showed that there was no practical and electrophysiological finding of neurotoxicity of HDR brachytherapy. Based on their clinical study [14], Zelefsky *et al*. speculated that the threshold tolerance of peripheral nerves to LDR brachytherapy might be higher than to EBRT. Similarly, HDR brachytherapy may have a higher threshold tolerance than EBRT, attributable to different radioactive sources.

Despite the small number of patients, our findings suggest that HDR brachytherapy may not adversely affect peripheral nerve function in the treatment of STS with neurovascular involvement. Latency also needs to be considered in evaluation of radiation neuropathy. A long-term follow-up study of EBRT reported that the incidence of complications involving nerves increased with time after radiation and not all of the cases were detected at 5, or even 10 years after the treatment [20]. More clinical data with a large number of patients treated with HDR brachytherapy and longer follow-up periods are required to detect further long-term morbidity.

## Conclusion

In this study, there was no practical and electrophysiological finding of neurotoxicity of HDR brachytherapy. Despite the small number of patients, our encouraging results are valuable for limb-preserving surgery of unmanageable STS involving critical neurovascular structures.

## Competing interests

The author(s) declare that they have no competing interests.

## Authors' contributions

TK conceived of the study, collected and analysed the data, and drafted the manuscript. TS, SS, TM, KH, and MO participated in the study design and manuscript preparation. HK carried out the electrophysiological study and a critical review of the manuscript. MK performed brachytherapy and a critical review of the manuscript. All authors read and approved the final manuscript.

## Pre-publication history

The pre-publication history for this paper can be accessed here:



## Figures and Tables

**Table 1:** Patients' clinicopathological characteristics

No	Sex	Age (years)	Histology	Site	Preserved nerve	Tumor grade	Size (cm)
1	Female	78	Myxoid liposarcoma	Forearm	Median, Radian, Ulnar	High	13.0
2	Male	48	Malignant fibrous histiocytoma	Thigh	Sciatic	High	5.5
3	Female	48	Myxoid liposarcoma	Forearm	Median	High	5.5
4	Male	35	Malignant fibrous histiocytoma	Thigh	Sciatic	High	9.0
5	Male	61	Extraskeletal chondrosarcoma	Pople	Popliteal	Low	10.0
6	Female	15	Synovial sarcoma	Buttock	Sciatic	High	10.0
7	Male	84	Malignant fibrous histiocytoma	Buttock	Sciatic	High	13.0

**Table 2:** Treatment results

No.	Margin	PTV^a ^(cm^3^)	Brachytherapy (Gy)	EBRT^b ^(Gy)	Failure	Complication	Follow-up (months)
1	Negative	65	50	-	-	Motor paresis	AWD^c ^(77)
2	Negative	63	50	(50)	Local	Fracture	AWD (75)
3	Negative	52	50	-	-	-	AWD (62)
4	Negative	93	50	-	-	-	AWD (43)
5	Negative	64	30	20	-	Sensory loss	AWD (34)
6	Negative	60	50	-	Lung	-	DOD^d ^(32)
7	Positive	134	50	-	-	-	DOC^e ^(13)

PTV^a ^= Planning target volume, EBRT^b ^= External beam radiation therapy, AWD^c ^= Alive without disease, DOD^d ^= Dead of disease, DOC^e ^= Dead from other cause

**Table 3:** Electrophysiological results

No.	Preserved nerve	Motor nerve or F-wave conduction velocity (m/s)		Months after brachytherapy
			
		Involved limb	Uninvolved limb	
2	Sciatic nerve	50.3	52.3	75
3	Median nerve	55.0	56.0	62
4	Sciatic nerve	58.5	50.1	43
